# Hydrogen Sulphide Treatment Increases Insulin Sensitivity and Improves Oxidant Metabolism through the CaMKKbeta-AMPK Pathway in PA-Induced IR C2C12 Cells

**DOI:** 10.1038/s41598-017-13251-0

**Published:** 2017-10-16

**Authors:** Xubo Chen, Xueyan Zhao, Fazhang Lan, Tao Zhou, Hua Cai, Haiying Sun, Weijia Kong, Wen Kong

**Affiliations:** 1 Department of Otorhinolaryngology, Union Hospital, Tongji Medical College, Huazhong University of Science and Technology, Wuhan, 430022 China; 2 Department of Endocrinology, Union Hospital, Tongji Medical College, Huazhong University of Science and Technology, Wuhan, 430022 China; 3 Department of Otorhinolaryngology, Southwest Hospital, Third Military Medical University, Chongqing, 400038 China

## Abstract

Studies have reported attenuation of insulin resistance (IR) by improving phosphorylation of the insulin signalling pathway. However, the upstream molecular signalling pathway is still elusive. In this study, Western blot was used to evaluate the phosphorylation level of the insulin signalling pathway and the AMPK pathway. 2-NBDG was used to evaluate glucose uptake. Ca^2+^ imaging was used to assess change of intracellular Ca^2+^ concentration. We found that NaHS enhanced the intracellular Ca^2+^ concentration and glucose uptake and activated the insulin signalling cascade in a palmitic acid (PA)-induced IR model in C2C12 cells. Furthermore, activation of the IRS1/PI3K/AKT pathway and glucose uptake were decreased when AMPK or CaMKKβ was inhibited. Our study also showed that the mitochondrial electron transport chain, ATP production, and intramitochondrial cAMP declined in the IR model but that this effect was reversed by NaHS, an effect that may be mediated by the Ca^2+^/CaMKK2/AMPK and PI3K/AKT pathways. Our data indicate that H_2_S improves activation of the insulin signalling cascade and glucose uptake via activation of the Ca^2+^/CaMKK2/AMPK pathway and mitochondrial metabolism in C2C12 cells. Furthermore, NaHS protects mitochondrial function and maintains normal ATP production by activating the cAMP system and the Ca^2+^/CaMKK2/AMPK and PI3K/ATK pathways.

## Introduction

Diabetes mellitus (DM) is a common clinical metabolic disorder characterised by hyperglycaemia as a result of insulin resistance (IR) and/or beta-cell failure. IR has a critical role in the pathogenesis of type 2 diabetes, which is characterised by decreased sensitivity to insulin and impaired glucose uptake. In obese people with insulin resistance, high levels of free fatty acids, including PA, are frequently observed in the clinic^1^. PA is closely related with occurence of IR in humans^2^ and commonly used to artificially induce insulin resistance in cultured cells^3^ due to its ability to activate inflammatory responses, such as the NFκB pathway, and to induce overproduction of reactive free radicals^4^.

Hydrogen sulphide (H_2_S), a powerful gaseous molecule that participates in diverse biological processes, has been used in clinical disease prevention or treatment, such as mechanical lung injury induced by ventilator. A study in an animal model has reported that hydrogen sulphide decreased mechanical ventilation-induced histological damage, inflammation and expression of stress proteins in lungs^5^. In addition, H_2_S is closely correlated with diabetes, though evidence of both pro-diabetogenic and anti-diabetogenic effects of H_2_S is present in the published literature. Some researchers have reported that higher expression levels of H_2_S and cystathionine γ-lyase (CSE) were associated with hypoinsulinaemia and hyperglycaemia in Zucker diabetic rats and that the inhibition of H_2_S restored serum insulin levels^6^, which suggested an alleviating effect of H_2_S on diabetes. In contrast, other researchers have reported that diabetic models treated with NaHS decreased oxidative stress^7^ and increased glucose uptake^8^. Moreover, serum H_2_S levels were increased and IR was alleviated after chronic feeding with garlic, which contains H_2_S donor substances^9^, suggesting a preventive effect of H_2_S on diabetes. Thus, the mechanism of action of H_2_S on the pathogenesis of DM remains unknown.

Experimental evidence also indicates that the pathogenesis of IR and diabetes is closely related with AMP-activated protein kinase (AMPK) activity^10,11^. AMPK activation blocks glucose induced insulin secretion in beta cells. In contrast, AMPK inactivation has been shown to lead to unregulated insulin secretion from MIN6 cells^12^. Furthermore, treatment of C2C12 muscle cells with an AMPK agonist had an additive effect on promoting IRS1-associated PI3K activity^13^. However, the potential mechanisms by which AMPK mediates the pathogenesis of IR and diabetes are still unclear.

Ca^2+^/calmodulin-dependent protein kinase 2 (CaMKKβ), a member of the Ca^2+^/CaM-dependent protein kinase family, is involved in coordinating many physiological and pathophysiological processes, such as energy balance^14^, glucose homeostasis^15^, inflammation^16^ and cancer^17^. Many studies have reported that CaMKKβ is an upstream kinase of AMPK^18,19^. Woods and co-workers^20^ reported that overexpression of CaMKKβ could increase AMPK activity and that conversely, inhibition of CaMKKβ activity by STO-609 or downregulation of CaMKKβ by RNA interference could decrease AMPK activation. Together, these data indicate that the CaMKKβ-AMPK pathway may interact with insulin sensitivity and glucose uptake. However, the specific mechanism is not fully understood.

In this study, we examined the influence of H_2_S on the insulin signalling pathway and the activity of AMPK and CaMKKβ in C2C12 mouse skeletal muscle cells treated with palmitic acid. We used this system to explore the underlying signalling mechanisms by which H_2_S affects IR. These data might shed light on mechanisms to improve IR and ameliorate metabolic disorders in diabetic patients in the future.

## Materials and Methods

### Reagents

Dulbecco’s modified Eagle’s medium (DMEM), penicillin/streptomycin (P/S) and trypsin were purchased from HyClone (Logan, UT, USA). Foetal bovine serum (FBS) was from Gibco BRL (Grand Island, NY, USA). Hydrogen sulphide hydrate was from Aladdin Industrial Corporation (Shanghai, China). Palmitate, compound C (CC), 2-[N-(7-nitrobenz-2-oxa-1,3-diazol-4-yl) amino]-2-deoxy- D-glucose (2-NBDG) and STO-609 were obtained from Sigma-Aldrich (St Louis, MO, USA). Fatty acid-free bovine serum albumin (BSA) and protease inhibitor cocktail were obtained commercially from Roche (Basel, Switzerland). The calcium indicator Fluo-3/AM was from Calbiochem (Darmstadt, Germany). Information about the antibodies we used is presented in Table 1. The specificity and sensitivity of all the antibodies used were tested and guaranteed as indicated in the manufacturers’ instructions.

**Table 1:** Information about the antibodies.

Peptide/Protein Target	Name of Antibody	Manufacturer; Catalogue No.	Host Species, Monoclonal or Polyclonal	Dilution
CBS	Anti-CBS antibody	Abcam; ab135626	Rabbit polyclonal	1:1000
MPST(D-8)	MPST antibody (D-8)	Santa Cruz Biotechnology; sc-374326	Mouse monoclonal	1:1000
CSE	CSE monoclonal antibody	Proteintech; 60234-1-lg	Mouse monoclonal	1:500
Actin	Anti-beta-actin monoclonal antibody	Lianke; Mab1445	Mouse monoclonal	1:3000
IRS1 (phospho Ser-312)	IRS1 (phospho Ser 312) antibody	GeneTex; GTX24865	Rabbit polyclonal	1:1000
IRS1	Anti-IRS1 antibody	Millipore; 04-784	Rabbit monoclonal	1:1000
Phospho-PI3 kinase p85 (Tyr-458)/p55 (Tyr-199) antibody	Phospho-PI3 kinase p85 (Tyr458)/p55 (Tyr199)	Cell Signaling Technology; 4228	Rabbit polyclonal	1:1000
PI3K	P85;PI3R1 monoclonal antibody	Proteintech; 60225-1-Ig	Mouse monoclonal	1:1000
AKT (phospho Ser-473)	AKT1 phospho (pS473) Rabbit monoclonal antibody	Epitomics; 2118-1	Rabbit monoclonal	1:1000
AKT	AKT 1 + 2 + 3 antibody	GeneTex; GTX121937	Rabbit polyclonal	1:4000
phospho-AMPK (Thr-172)	p-AMPKα1/2 Antibody	Santa Cruz Biotechnology; sc-33524	Rabbit polyclonal	1:600
AMPKα_1_/α2	AMPKα_1_/AMPKα_2_ (ab-174/ab-172) antibody	Signalway Antibody; 21191	Rabbit polyclonal	1:1000
OXPHOS	Anti-Rt/Ms Total Oxphos Complex Kit	lifetechnologies	Mouse monoclonal	1:1000

### Cell culture and treatment

Mouse C2C12 skeletal muscle myoblast line was obtained from Wuhan University (Wuhan, Hubei, China). Cells were incubated in a culture flask, 12-well plate or confocal basin in a humidified atmosphere of 5% CO_2_ at 37 °C in DMEM containing 10% FBS and 1% P/S, with 0.25% trypsin used to passage cells. Differentiation of C2C12 myoblasts was performed by incubating cells in fresh DMEM containing 1% P/S, 0.1% FBS and 50 nM insulin for 4 days.

For the palmitate-induced IR model, we used the procedure previously described by Wang *et al*.^21^. C2C12 myotubes were starved in serum-free DMEM for 5 hours before incubating with 0.35 mM palmitate for 18 hours. Palmitate stock solutions (0.8 mM) were prepared in 20% fatty acid-free BSA at 56 °C and then diluted in culture medium to a final concentration of 0.35 mM.

### Cell viability test

Healthy cells were seeded on a 96-well flat-bottomed plate at a density of 2 × 10^3^ cells per well and then incubated overnight before induction of IR as described above. The medium was replaced with 100 μl of fresh medium, and 10 μl of CCK-8 dye solution was added to each well, which was followed by a 1-hr incubation at 37 °C. Absorbance was measured at 450 nm by a microplate spectrophotometer (ELX800, BioTek Instruments Inc, Winooski, VT, USA).

### Western blot analysis

After induction of IR, protein lysates were extracted. Following three washes in ice-cold PBS, RIPA lysis buffer (Beyotime Biotech, Haimen, Jiangsu, China) containing 1% phenylmethylsulphonyl fluoride (PMSF; Beyotime, Haimen, Jiangsu, China) and 3% phosphatase inhibitor cocktail tablets were added. Protein extracts (8 μg or 10 μg) were subjected to SDS-PAGE on 8% gels and then transferred to polyvinylidene difluoride (PVDF) membranes (EMD Millipore, Billerica, MA, USA) by electro-blotting. The membranes were blocked with 5% non-fat milk for 1 hour before incubation with primary antibodies overnight at 4 °C. The targets of the primary antibodies included the following: Cystathionine β-synthase (CBS), 3-mercaptopyruvate sulphurtransferase (MPST), CSE, phosphorylated Insulin Receptor Substrate Protein 1 (p-IRS1), phosphorylated Phosphatidylinositol 3-kinase (p-PI3K), p-AKT, p-AMPK and Oxidative Phosphorylation (OXPHOS) complexes. After incubation with HRP-conjugated secondary antibodies and Western blot lightning ECL exposure, all PVDF membranes of phosphorylated antibodies were treated with stripping buffer (Beyotime Biotech, Haimen, Jiangsu, China). Next, the stripped PVDF membranes were blocked in 5% non-fat milk and used for the corresponding total antibodies, including IRS1, PI3K, AKT and AMPK. The grey scale intensity was obtained using Adobe Photoshop CS3 software (Adobe Systems Inc, San Jose, CA, USA).

### Glucose uptake

Glucose uptake was assayed using 2-NBDG as described previously^22^ with minor modifications. C2C12 myotubes were grown in confocal basins. 2-NBDG was diluted in DMSO to a concentration of 2 mg/ml and stored at −20 °C. After treatment with H_2_S or CC + H_2_S or STO + H_2_S, cells were incubated with or without media containing 10 nM insulin for 10 minutes. The media was then changed to low-glucose DMEM containing 150 μg/ml 2-NBDG for an additional 60 minutes at 37 °C. The medium was removed, and cells were washed 3 times with cold PBS. Cells were then transferred to black 96-well plates after trypsinisation. 2-NBDG uptake was measured using a fluorescence microplate reader (BioTek Instruments, Winooski, VT, USA).

### Calcium imaging

C2C12 myotubes were incubated with 5 μM Fluo-3/AM for 40 min at 37 °C to detect a change in the intracellular Ca^2+^ levels. After incubation, the myotubes were washed two times and incubated in Tyrode’s solution^23^ with 1.8 mM CaCl_2_. Images of calcium influx were obtained using an inverted confocal laser scanning microscope (Nikon, Japan). Cells were excited with an argon laser at a wavelength of 488 nm and observed with a 10× objective and 2× optical zoom for a total magnification of 20×. Images were scanned at a frame rate of 3 seconds per frame, and 600 frames were obtained to record a total of 30 min per stimulation. During the acquisition, NaHS (50 µM) was added after 160 seconds. The acquisition software generated the plotted results.

### Measurement of ATP production

An ATP Assay Kit (S0026, Beyotime, Haimen, China) was used to determine the content of ATP in cultured C2C12 cells according to the manufacturer’s instructions. The chemiluminescence signal was read with a Multi-Mode microplate reader (BioTek Instruments, Winooski, VT, USA). Data were normalized for protein content.

### Measurement of ADP/ATP ratio

The ratio of Adenosine-5′-diphosphate (ADP)/Adenosine- 5′-triphosphate (ATP) was measured by an ADP/ATP Luminescent Cell Viability Assay Kit (TIANDZ, Beijing, China) with a Multi-Mode Microplate Reader according to the manufacturer’s instructions.

### Mitochondria isolation and measurement of mitochondrial cAMP levels

After the indicated treatments of C212 cells were finished, mitochondria were prepared using a mitochondria isolation kit according to the handbook (Qiagen, NewYork, USA). Mitochondria were lysed with 0.25% Triton-X regent. The Cyclic Adenosine Monophosphate (cAMP) was measured by a cAMP Assay Kit (Nanjing Jiancheng Biotec, Nanjing, China) according to the instructions with a Multi-Mode Microplate Reader at 405 nm. Serial dilutions of cAMP were used as calibration standards. Data were normalized for protein content.

### Statistical analysis

All experiments were performed at least three times, with the results presented as the mean ± SEM using SPSS 12.0 software (SPSS Inc., Chicago, IL, USA). Statistical comparisons were analysed by one-way ANOVA unless otherwise noted. Significance was considered at a probability error (p) < 0.05, and all p values were two-tailed.

## Results

### Glucose uptake and expression of p-AKT are impaired in the PA-induced IR cell model

**Figure 1:**
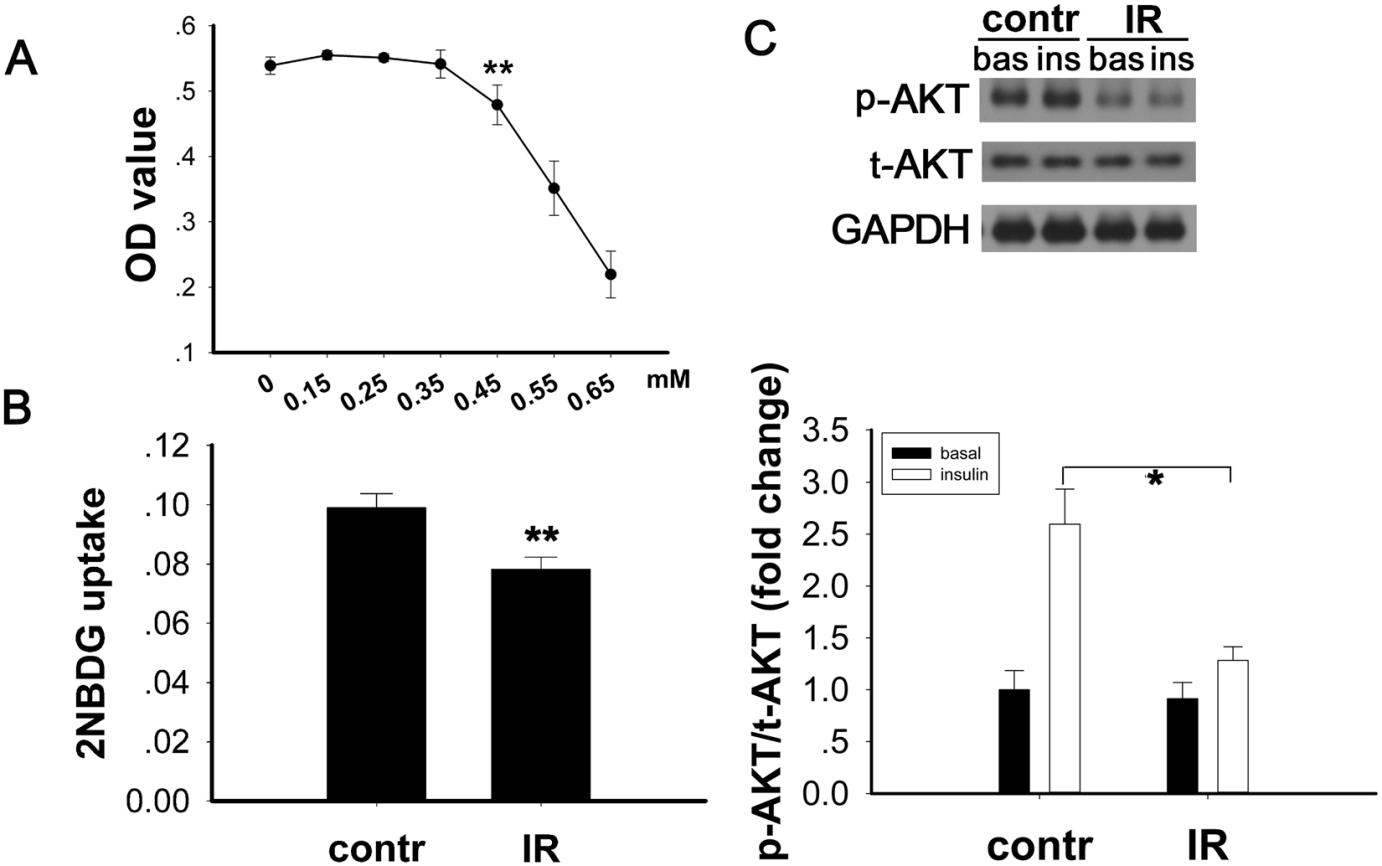
Impairment of glucose uptake by PA in the cellular IR model. (**A**) An effective concentration of PA was determined by CCK-8 test. Differentiated C2C12 cells were starved in serum-free DMEM for 5 hours. Next, the cells were cultured in DMEM (low glucose) containing 150 μg/ml 2-NBDG for 1 hour at 37 °C. (**B**) Glucose uptake was decreased, and (**C**) the expression of p-AKT was decreased after PA treatment. Data are shown as the mean ± sem. *p < 0.05 vs. control, **p < 0.01 vs. control.

A cellular model of insulin resistance was induced by treatment of C2C12 myotubes with palmitic acid (PA). The appropriate concentration of PA was determined based on the results of a CCK-8 cell viability test (Fig. 1A). Differentiated C2C12 cells were treated with either PA conjugated to 20% BSA (IR group) or 20% BSA alone (control group) for 18 hours. In the PA-induced IR model, insulin-stimulated glucose uptake was lower than in control cells (Fig. 1B, p < 0.001). This result suggested that insulin resistance was induced by the treatment with PA. To investigate whether the insulin signalling pathway was impaired, we evaluated the phosphorylation level of AKT (Ser-473) by Western blot. Compared to the control, both the basal and insulin-stimulated AKT phosphorylation were downregulated after treatment with PA (Fig. 1C, p < 0.05).

### Enzymes involved in production of endogenous H_2_S were decreased in the PA-induced insulin resistance model

**Figure 2:**
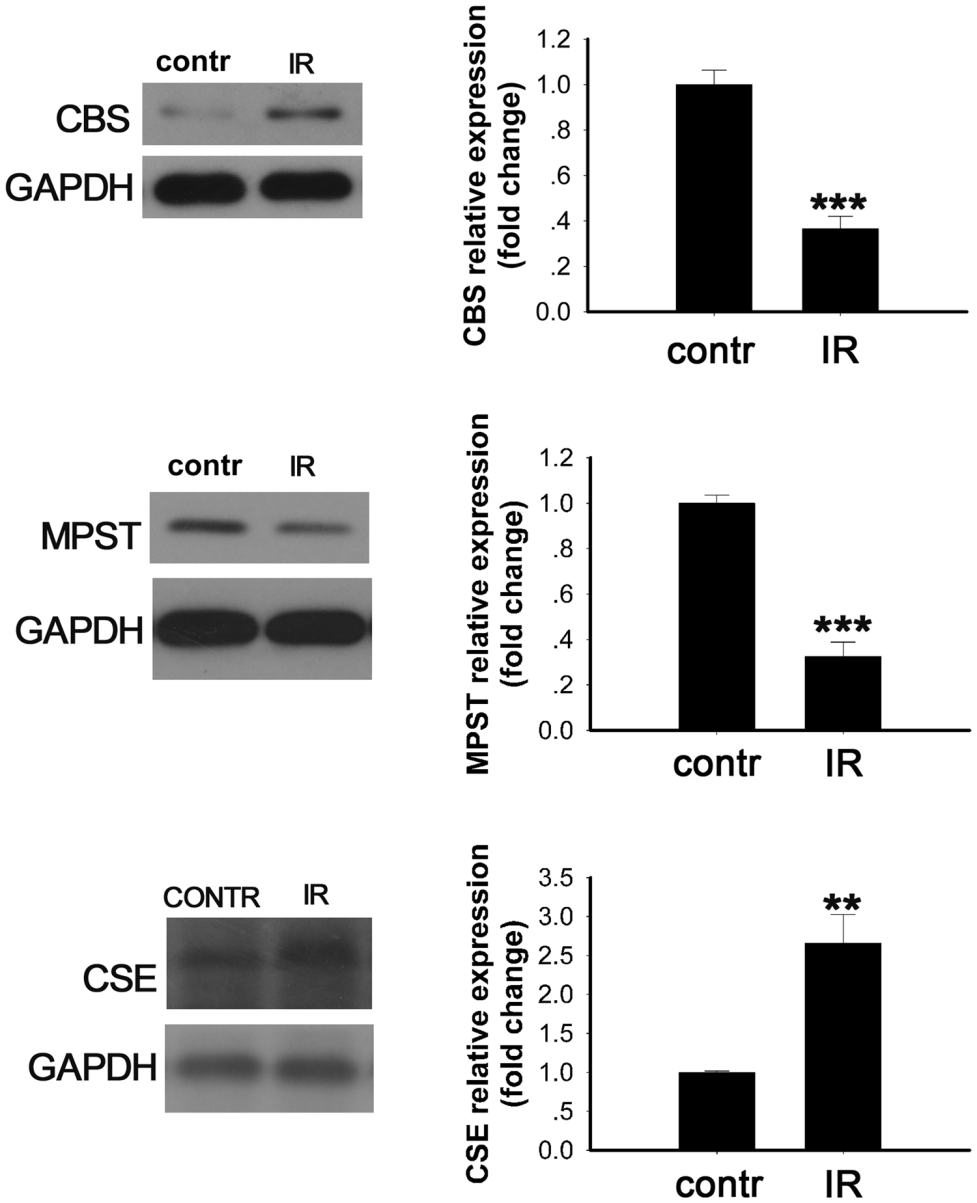
Enzymatic changes related to endogenous H_2_S production in the PA-induced insulin resistant cell model. Expression levels of the indicated enzymes were evaluated by Western blot: (**a**) CBS, (**b**) MPST, and (**c**) CSE. Data are shown as the mean ± sem. An independent sample t-test was performed to compare the difference between the control and IR groups. **p < 0.05 vs. control, ***p < 0.01 vs. control.

CBS, MPST and CSE are three enzymes involved in endogenous H_2_S generation. We evaluated the expression of these enzymes in the PA-treated C2C12 cells by Western blot (Fig. 2). The expression of CBS and MPST decreased in the PA-treated group compared with the control group (Fig. 2a and b, p < 0.001 and p < 0.001 vs. control, respectively). However, the expression of CSE was upregulated in the PA-treated group compared with the control group (Fig. 2c, p < 0.01 vs. control).

### Exogenous NaHS promotes glucose uptake and enhances phosphorylation of the insulin signalling pathway in normal C2C12 cells and in the IR cell model

**Figure 3:**
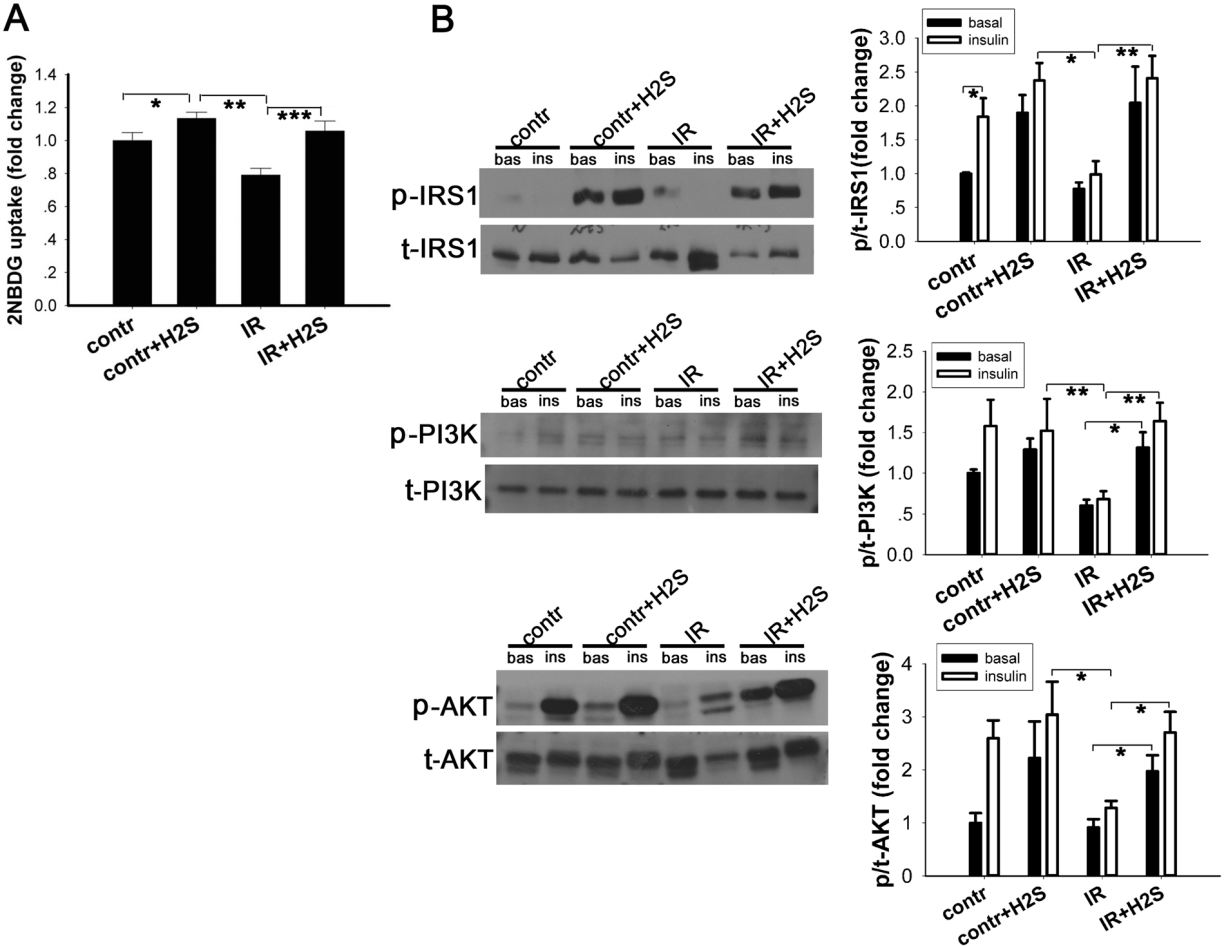
Exogenous NaHS promoted glucose uptake and increased the phosphorylation of insulin signalling pathway components in C2C12 cells. After insulin resistance was induced by PA, culture medium was completely removed and replaced with NaHS solution. NaHS solution was freshly prepared and diluted to 50 μM with DMEM. (**A**) Cells were incubated in NaHS solution for 30 minutes at 37 °C, followed by incubation with 10 nM insulin and 150 μg/ml 2-NBDG. NaHS treatment promoted glucose uptake in both control C2C12 cells and the IR cell model. (**B**) Phosphorylation levels of IRS1, PI3K and AKT were evaluated by Western blot. In the PA-treated group, phosphorylation of IRS1, PI3K and AKT were decreased. Administration of NaHS increased the phosphorylation of IRS1, PI3K and AKT. *p < 0.05, **p < 0.01, ***p < 0.001. Data are shown as the mean ± sem.

As the expression of enzymes involved in H_2_S production were changed in the IR cell model, we hypothesised that increasing H_2_S artificially may attenuate the IR induced by PA. Sodium hydrosulphide (NaHS) is a well-known and widely used donor of H_2_S. NaHS was freshly dissolved and used immediately for each experiment. A previous study found that exogenously applied H_2_S at 50–200 μM reduced INS-1E cell viability in a dose-dependent manner. However, the cell viability with 50 μM H_2_S treatment was decreased less than 20% that in the absence of H_2_S^24^. In addition, it was reported that in myotubes and adipocytes, the viability of cells were decreased significantly at the concentration above 500 μM when NaHS exposured for 24 hours. Morerover, Xue’s study showed that NaHS-induced (50 μM) increase in insulin receptor (the upstream factor of insulin signaling cascade) phosphorylation occurred from 5 to 240 min in adipocytes but not in time-dependent manner^8^. Therefore, we choose the concentration of NaHS at 50μmol/L and exposure time at 30 minutes. After incubation with NaHS (50 μM, 30 minutes), glucose uptake was improved in both the IR group (p < 0.001 vs. cells pretreated with PA but without NaHS) and in the normal control group cells (p < 0.001 vs. normal control cells not treated with NaHS) after stimulation with 10 nM insulin for 10 minutes (Fig. 3A). These results indicate NaHS incubation can have beneficial effects to insulin sensitivity. To determine potential mechanisms mediating these effects, we incubated the C2C12 myotubes with 50 μM NaHS for 30 min and then stimulated with 10 nM insulin for an additional 10 min. Then, we analysed the phosphorylation and corresponding total protein levels of the insulin signalling pathway. As shown (Fig. 3B), incubation with PA caused decreased phosphorylation of the IRS1/PI3K/AKT pathway whether the cells were stimulated with or without insulin. After administration with exogenous NaHS (50 μM, 30 min), the phosphorylation of IRS1 (Ser-312), PI3K (Tyr-458) and AKT (Ser-473) were increased in both control group cells and IR group cells, particularly after insulin stimulation (10 nM, 10 min).

### Exogenous NaHS increased intracellular concentration of calcium ions

**Figure 4:**
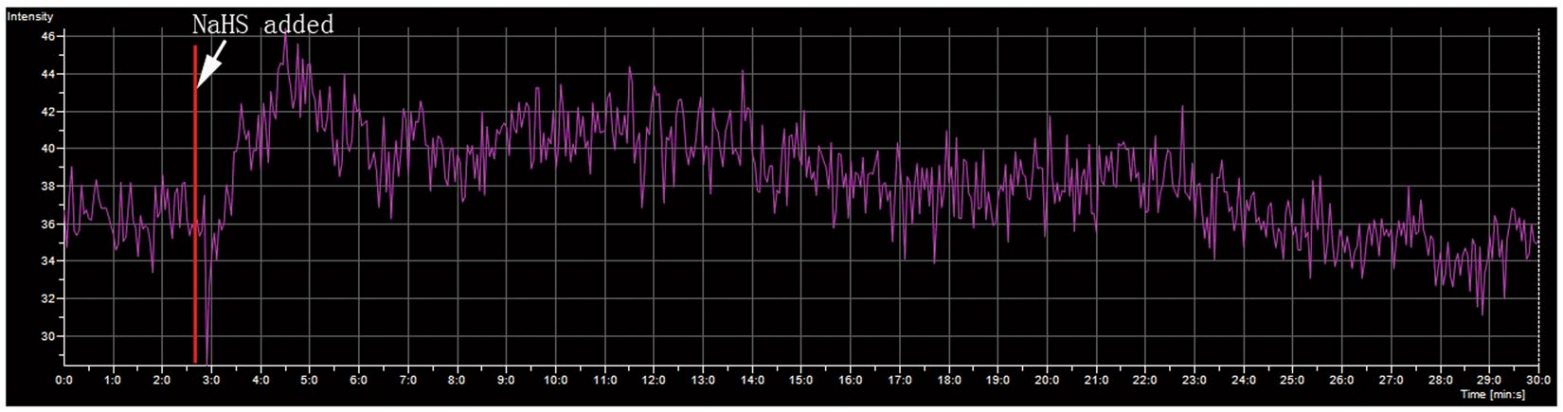
Exogenous NaHS impacts cytoplasmic calcium ion concentrations. C2C12 myotubes were incubated with Fluo-3/AM (5 μM) for 40 min. After washing twice with PBS, the cells were incubated in HBSS with 1.8 mM CaCl_2_. The intensity of Fluo-3 was obtained before and after NaHS was added (indicated by red line and white arrow), with the Fluo-3 acquisition lasting for 30 min. The fluorescence intensity of Fluo-3/AM was rapidly enhanced when NaHS was added and then slowly decreased to baseline.

The calcium signal plays a crucial role in the regulation of glucose metabolism, such as mediating glucose transport in skeletal muscle^25^. In this study, we determined the intracellular Ca^2+^ concentration in C2C12 cells by the indicator Fluo-3/AM. C2C12 myoblasts were seeded in confocal basins. After differentiation into myotubes, we stimulated with NaHS and measured the change in intracellular Ca^2+^. Extrogenous NaHS was added at the 160th second. As the data show (Fig. 4), the intracellular concentration of Ca^2+^ was increased immediately after NaHS was added (50 μM, working concentration) in the group of PA-induced IR myotubes.

### Exogenous NaHS elevated the AMPK phosphorylation and the ratio of ADP/ATP in a PA-induced IR cell model

**Figure 5:**
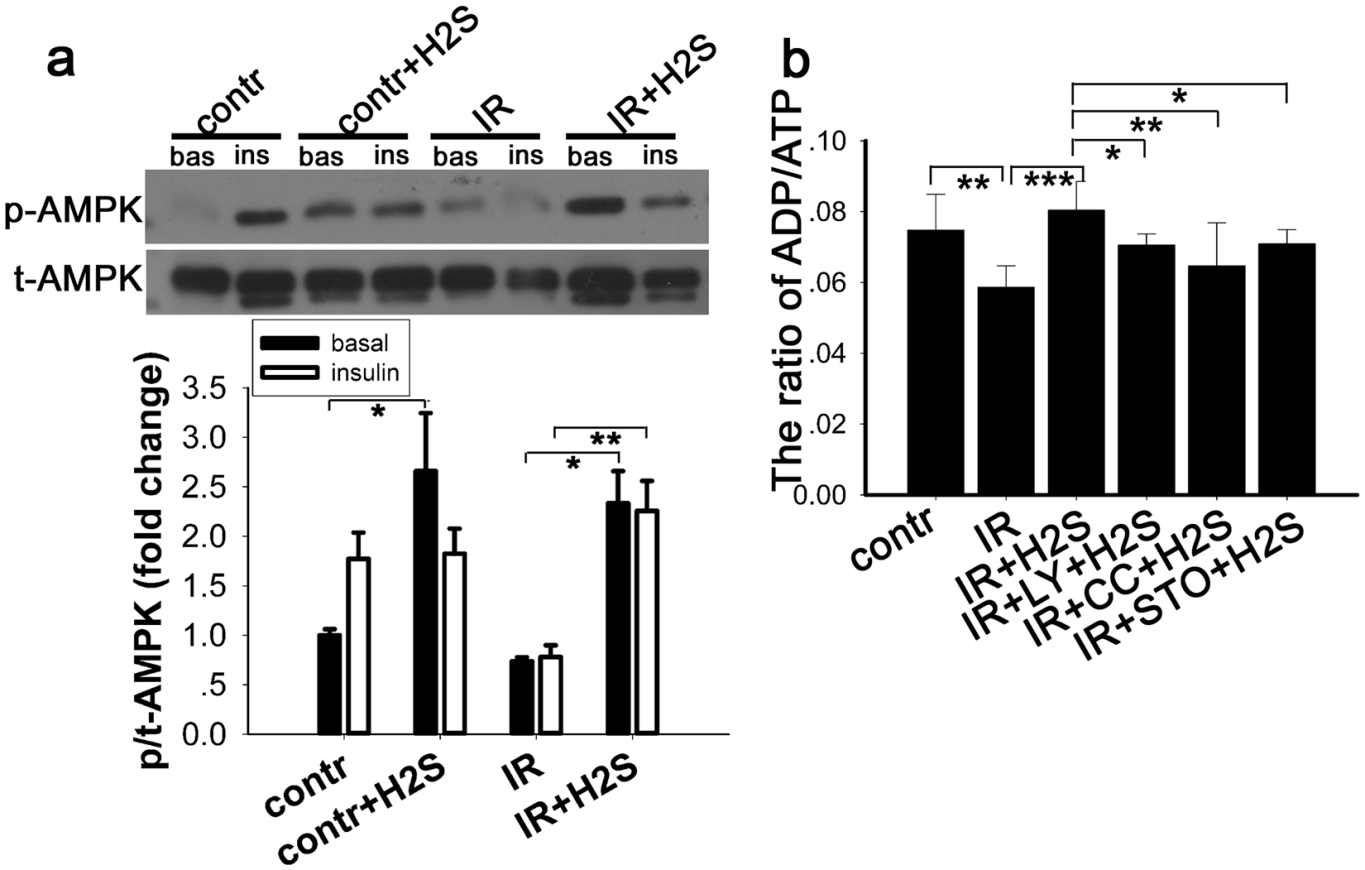
AMPK activation by exogenous NaHS in normal C2C12 myotubes and in the IR cell model. (**a**). C2C12 myotubes were co-cultured with 50 μM NaHS for 30 min and then stimulated with or without 10 nM insulin for 10 min. Cell lysates were prepared and the phosphorylation level of p-AMPKα1/2 (Thr-172) was evaluated by Western blot. Phosphorylation of AMPK was increased after treatment with NaHS. *p < 0.05, **p < 0.01, ^#^p < 0.001. Data are shown as the mean ± sem. (**b**) Ratio of ADP/ATP decreased in IR cells and this effect was reversed by NaHS. The effect of NaHS on ADP/ATP was diminished by CamKK2, AMPK and PI3K inhibitors. *p < 0.05, **p < 0.01, ***p < 0.001. Data are shown as the mean ± SD.

It has been reported that AMPK plays a pivotal role in glucose uptake as well as in modulating appetite^26^, and so we also examined the phosphorylation of AMPK in C2C12 myotubes preincubated with NaHS (50 μm, 30 min). AMPK (Thr-172) phosphorylation levels were increased after stimulation with insulin (10 nM, 10 min) in control cells (Fig. 5a). In cells incubated with PA, the phosphorylation of AMPK was downregulated when compared with the control. Moreover, the downregulated AMPK phosphorylation due to IR induced by PA was significantly increased after exogenous NaHS administration. In addition, it has been reported that the ratio of ADP/ATP was closely related with AMPK phosphorylation^27^. Therefore, we tested the ADP/ATP ratio with an ADP/ATP Luminescent Cell Viability Assay Kit. The results showed that the ADP/ATP ratio is decreased in PA induced IR cells but that NaHS treatment increased the ADP/ATP ratio significantly (Fig. 5b). These observations suggested that the increased the phosphorylation of AMPK in the IR cellular model may be mediated by regulating the ADP/ATP ratio.

### The AMPK inhibitor, compound C, counteracted the effects of exogenous NaHS in the PA-induced IR cell model

**Figure 6:**
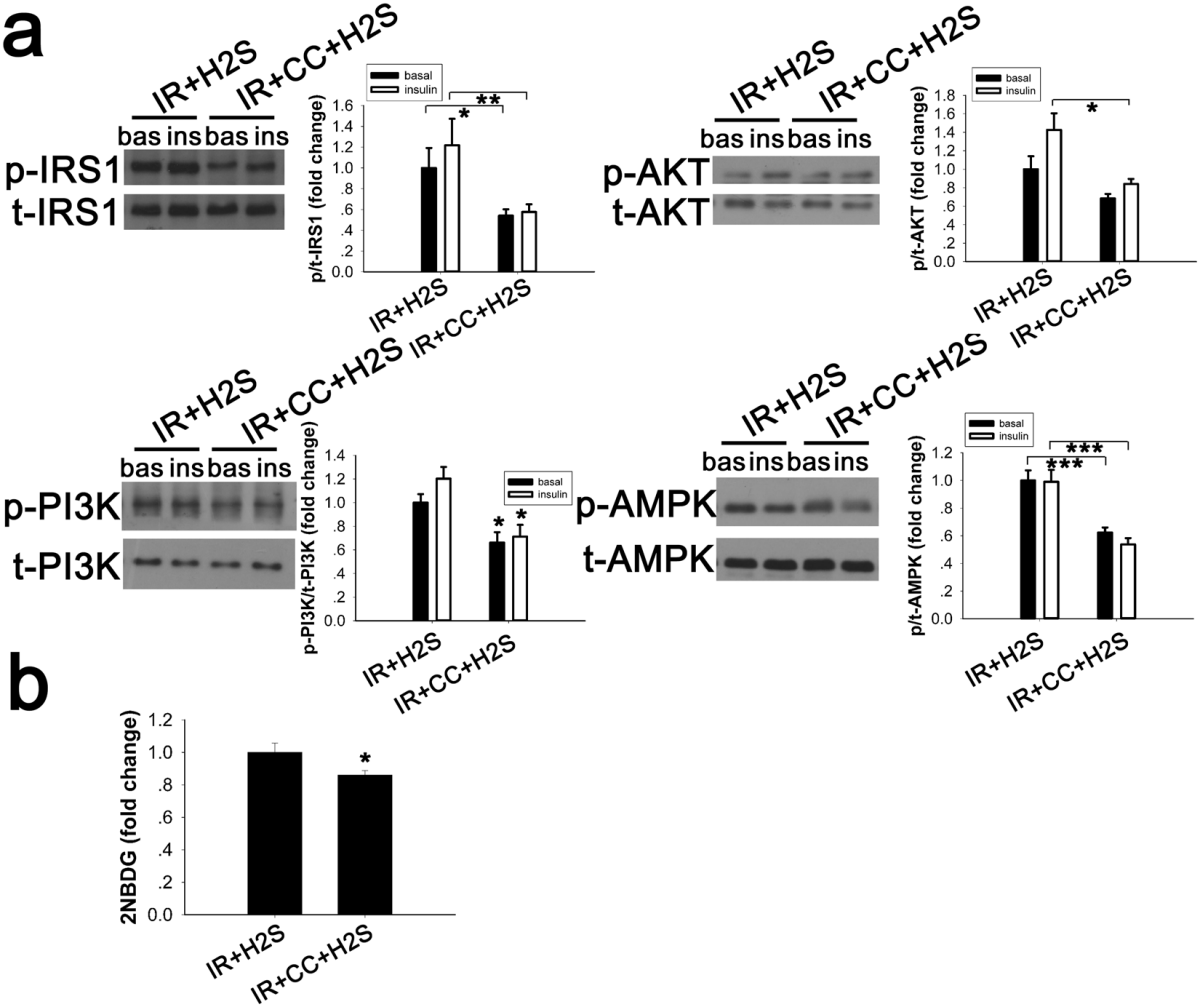
Compound C decreases insulin signalling and reduced NaHS-induced glucose uptake in IR cells. PA-treated C2C12 cells incubated with compound C (20 μM, 30 min) were cultured with DMEM containing 50 μM NaHS for 30 min. (**a**) NaHS-induced phosphorylation of IRS1, PI3K, AKT and AMPK was blocked by compound C. (**b**) NaHS-induced enhancement of glucose uptake was inhibited by compound C (20 μM, 30 min) in IR cell models. *p < 0.05, **p < 0.01, ***p < 0.001. Data are shown as the mean ± sem.

After confirming that NaHS could improve glucose uptake and activate the IRS1/PI3K/AKT pathway and AMPK, we examined whether the beneficial effects of NaHS were due to the activation of the AMPK-IRS1/PI3K/AKT pathway. In our study, compound C was dissolved in dimethylsulphoxide (DMSO), which is a common organic solvent. DMSO can induce cytotoxicity^28,29^, and all the comparison groups were treated with an equal concentration of DMSO (2 μl/ml). As our data show (Fig. 6a), cells preincubated with compound C (25 μM, 30 min) showed substantial downregulation of IRS1, PI3K and AKT activity (vs. cells pretreated with NaHS but without compound C) with or without insulin stimulation. Meanwhile, glucose uptake was not improved in cells pretreated with compound C and NaHS (p < 0.001 vs. cells treated with NaHS but without compound C), which is consistent with the variation in the IRS1/PI3K/AKT pathway after treatment (Fig. 6b).

### CaMKKβ inhibitor blocked the upregulation of the IRS1/PI3K/AKT signalling pathway and AMPK phosphorylation induced by NaHS and reduced glucose uptake in insulin resistance cell model

**Figure 7:**
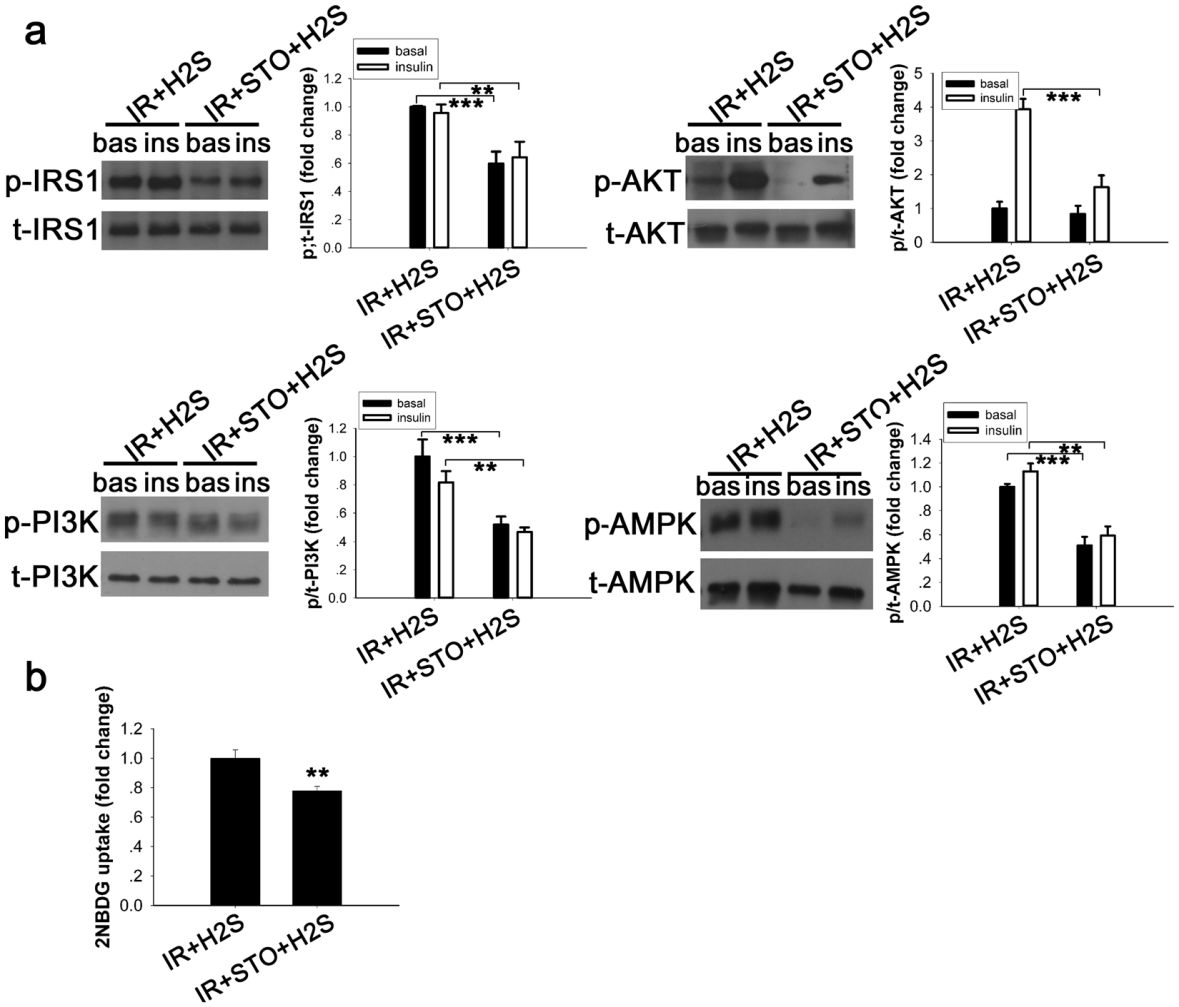
STO-609 inhibition of the effect of NaHS to stimulate the insulin signalling pathway and AMPK to improve glucose uptake in the IR cell model. (**a**) C2C12 myoblasts were treated with the CaMKKβ inhibitor, STO-609 (25 μM, 30 min), and stimulated with or without insulin (10 nM, 10 min). STO-609 blocked the phosphorylation of IRS1, PI3K, AKT and AMPK induced by NaHS. (**b**) Additional increases of the NaHS-induced 2-NBDG uptake was blocked by STO-609 (25 μM, 30 min) in PA-induced IR C2C12 cells. **p < 0.01, ***p < 0.001. Data are shown as the mean ± sem.

CaMKKβ, an important signalling molecule, is activated when the concentration of intracellular Ca^2+^ rises in response to the action of a hormone or drug. It has been reported previously that the Ca^2+^/calmodulin-activated protein kinase kinases, especially CaMKKβ, is one of the major upstream kinases of AMPK^20^. To determine whether the effects of NaHS could partly mediated by CaMKKβ, STO-609, a selective inhibitor of Ca2+/calmodulin-dependent protein kinase^18^ was used. As shown by the Western blot analysis (Fig. 7a), the phosphorylation of IRS1, PI3K, and AKT was reduced as well as the activity of AMPK, especially when stimulated with insulin. Correspondingly, the improvement in glucose uptake was blocked by the CaMKKβ inhibitor (Fig. 7b).

### Protective effects of NaHS on mitochondrial biogenesis were blocked by LY-294002, Compound C and CamKKβ in the IR cellular model

**Figure 8:**
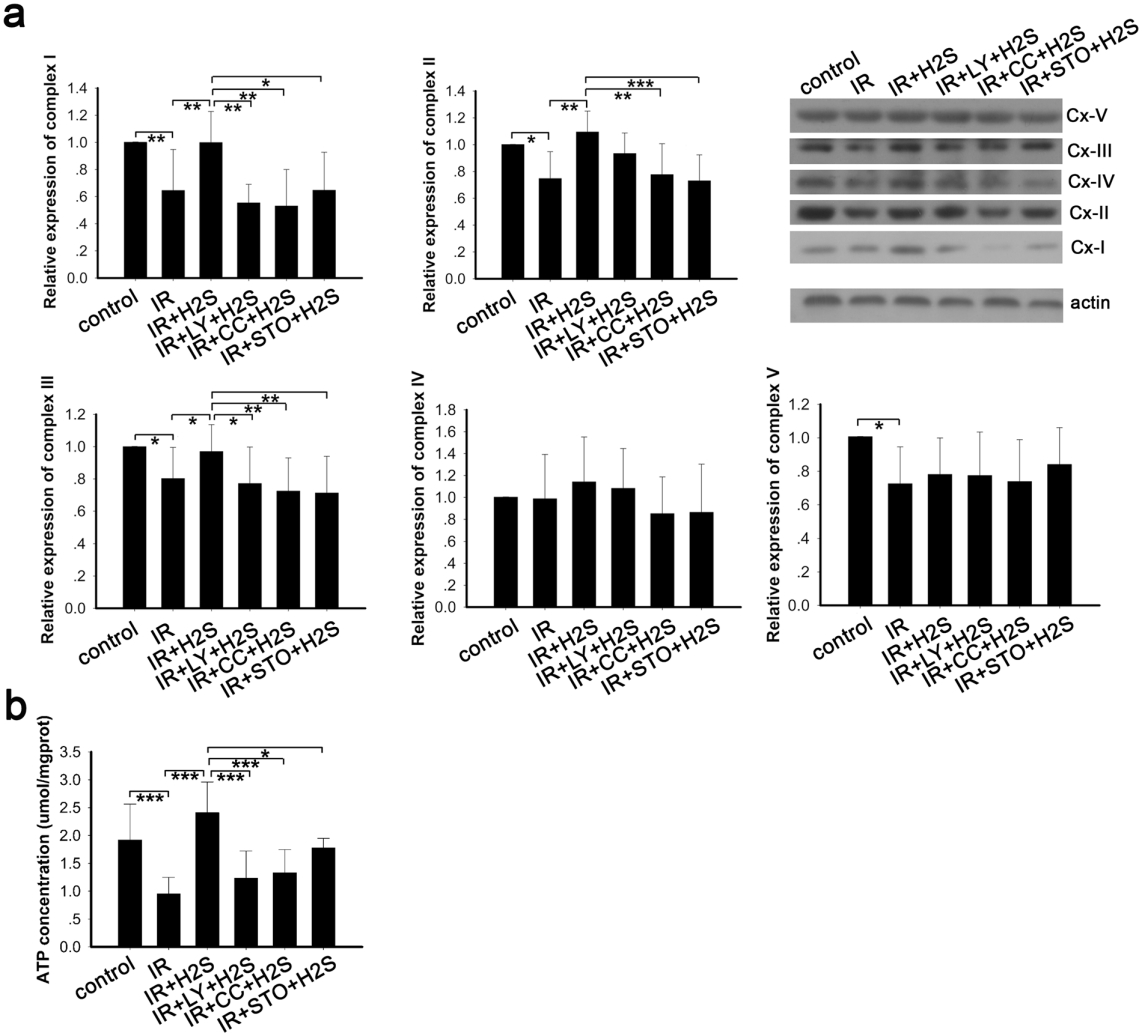
Protective effects of NaHS on mitochondrial biogenesis was blocked by LY-294002, Compound C and CamKKβ in IR cellular model. (**a**) The mitochondrial complexes were determined by Western blot. Expression of OXPHOS components was decreased in the PA-induced IR model and reversed by NaHS treatment. Inhibitors of CamKK2, AMPK and PI3K blocked the effects mediated by NaHS. *p < 0.05, **p < 0.01, ***p < 0.001, ^#^p < 0.05, ^##^p < 0.01, ^+^p < 0.05, ^++^p < 0.01, ^^p < 0.01. Data are shown as the mean ± SD. (**b**) ATP production in the IR group was decreased, but upregulated in response to NaHS treatment. Inhibitors of CamKK2, AMPK and PI3K blocked this upregulation. *p < 0.05, **p < 0.05, ***p < 0.001. Data are shown as the mean ± SD.

In eukaryotic cells, mitochondria are crucial organelles involved in energy metabolism and are called “energy factories”. In our study, we detected the expression of mitochondrial complexes and evaluated the mitochondrial function by detecting ATP production. Whole cell lysates were used to determine the expression levels of mitochondrial complexes using antibodies against the OXPHOS complex. Expression of mitochondrial complexes declined in the PA-induced IR cellular model (Fig. 8a). Meanwhile, NaHS improved the expression levels of mitochondrial complexes significantly, especially complexes I, II and III (Fig. 8a). However, pretreatment with LY-294002, Compound C and STO-609 inhibited the upregulation mediated by NaHS (Fig. 8a). ATP is mainly produced in the mitochondria, and the mitochondrial complexes play crucial roles in mitochondrial biogenesis. As our data show, ATP production is decreased in the PA-induced IR models *in vitro*, while NaHS preserved the mitochondrial biogenesis in the IR models (Fig. 8b). The inhibitor of CamKKβ blocked the preservation of mitochondrial biogenesis, but inhibitors of PI3K and AMPK did not (Fig. 8b). These data suggested that NaHS protected mitochondrial function and maintained normal mitochondrial biogenesis through CamKKβ, AMPK and PI3K pathways.

### Elevation of mitochondrial cAMP by NaHS was blocked by LY-294002, Compound C and CamKKβ in the IR cellular model

**Figure 9:**
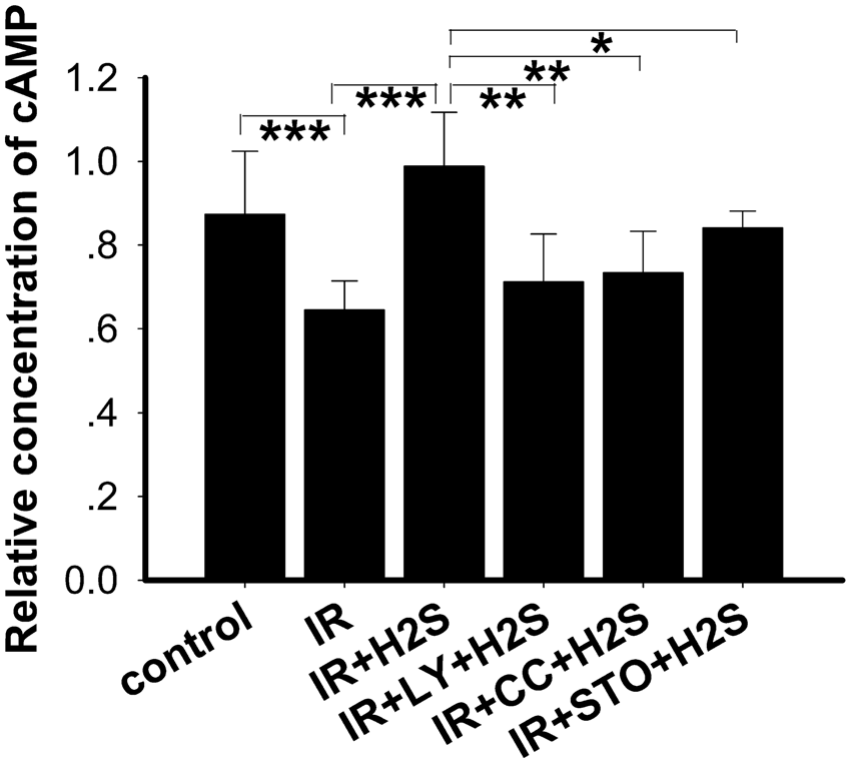
Elevation of mitochondrial cAMP by NaHS was blocked by LY-294002, Compound C and CamKKβ in the IR cellular model. The cAMP levels were measured in mitochondria. NaHS reversed the declined cAMP level of the IR model and this effect was blocked by inhibitors of CamKK2, AMPK and PI3K. *p < 0.05, **p < 0.01, ***p < 0.001. Data are shown as the mean ± SD.

We have tested the level of cAMP in mitochondria in PA-induced IR cells and evaluated the effect of NaHS on cAMP levels of mitochondria isolated from C2C12 cells. In our study, we found that the cAMP was decreased compared with the control group (Fig. 9), and that NaHS treatment of the C2C12 cells resulted in an elevation of cAMP levels in mitochondria (Fig. 9). Nevertheless, the elevations were blocked by inhibitors of PI3K, AMPK and STO-609 (Fig. 9). These observations suggested that the cAMP signalling pathway was downregulated in PA -induced IR cells and that NaHS administration reversed this change via the CamKKβ, AMPK and PI3K pathways.

## Discussion

CBS, MPST and CSE are three dominant enzymatic contributors to endogenous H_2_S production^30^. In the present study, we found that in the PA-induced cellular IR model, the expression of CBS and MPST were decreased. However, CSE expression was upregulated after treatment with PA. In different tissues, the major enzymes producing H_2_S can vary. CSE is the major synthetase of H_2_S in the cardiovascular system^31,32^ even though CSE is expressed in skeletal muscle in mammals^33^. Our observations indirectly suggest a decline in endogenous H_2_S production in insulin resistant cells. This observation is similar to a previous study in animals^34^. Our results also demonstrated that the glucose uptake was impaired in the PA-treated group, while it was improved after exogenous H_2_S was added, which may suggest a beneficial role of NaHS to alleviate insulin resistance. This supports previous *in vitro* and *in vivo* studies^8,35^.

AMPK is expressed in all eukaryotic cells, containing a catalytic α subunit and regulatory β and γsubunits^36^. The AMPK pathway is associated with glucose metabolism. Our study suggests that exogenous H_2_S supported by NaHS enhances the phosphorylation of AMPK in both the insulin resistant cell model and in the control cells. Correspondingly, glucose uptake, as evaluated by 2-NBDG, was increased. Studies by others have suggested that exercise and electrically stimulated contraction improves glucose uptake via induced phosphorylation of AMPK. Furthermore, our data show that the ratio of ADP/ATP is decreased in the PA-induced IR model *in vitro*, an effect that is reversed by NaHS treatment. The ratio of ADP/ATP is a key regulator of AMPK activity^27,37^. ADP binding to the γ-subunit induces a conformational change that protects AMPK from inactivation by phosphatases, and so AMPK acts as a sensor of the ADP/ATP ratio. In addition, 5-aminoimidazole-4-carboxamide riboside (AICAR), a widely used and cell-permeable activator of AMPK, stimulates glucose uptake in skeletal muscle *in vivo* and *in vitro* ^38,39^. These findings suggest that H_2_S ameliorates the impaired glucose uptake in an insulin resistance cell model by improving the activity of AMPK, which may respond by increasing the ADP/ATP ratio. Furthermore, our study also suggests that compound C, a pharmacological inhibitor of AMPK, inhibits the increased phosphorylation of AMPK signalling via the IRS1/PI3K/AKT pathway and the enhanced glucose uptake that were induced by NaHS. Some researchers have reported that exogenous H_2_S can activate the PI3K/AKT pathway by increasing insulin receptor sensitivity, leading to improved glucose uptake *in vitro* and *in vivo* ^8^. Insulin sensitivity can be regulated by serine/threonine phosphorylation of the insulin receptor substrate proteins IRS1 and IRS2^40^. Thus, in our study, the AMPK pathway and insulin signalling pathway may contribute to the improvements in glucose uptake following incubation with exogenous NaHS. Some authors also reported that AMPK rapidly phosphorylates IRS1 (Ser-789) in C2C12 myotubes pretreated with AICAR and induces an additional 65% increase in IRS1-associated PI3K activity when stimulated with insulin^13^. The results of our study combined with those of other researchers support a potential interaction between AMPK and insulin signalling.

As many studies reported that calcium ions (Ca^2+^) play a critical role in glucose uptake in skeletal^41,42^ muscle, we considered that Ca^2+^ may be involved in the regulation of insulin sensitivity. Our study demonstrates that exogenous H_2_S increases the Ca^2+^ concentration, activates the insulin signalling pathway and enhances glucose uptake in C2C12 cells. Some researchers have reported that exogenous H_2_S increased intracellular Ca^2+^ concentrations mainly via ryanodine receptors in rat parotid acinar cells^43^. Cells treated with STO-609, an inhibitor of CaMKKβ, showed decreased insulin signalling and AMPK phosphorylation induced by exogenous NaHS in the insulin resistance model. Furthermore, glucose uptake was not restored by NaHS when pretreated with STO-609. An earlier study reported that AMPK activated by either AICAR or increased cytosolic Ca^2+^ with caffeine promotes glucose uptake; however, when CaMKII was blocked with the inhibitors KN62 and KN93, the effect of caffeine on glucose uptake was also blocked^44^. Taken together, these findings suggest that a Ca^2+^/CaMKKβ-AMPK-IRS1/PI3K/AKT signalling system mediates the amelioration of IR induced by exogenous NaHS, at least in our cell model. As reported previously, CaMKKβ and AMPK participate in signal transduction associated with the mature bovine corpus luteum^45^, in the generation of androgen-related prostate cancer and in the migration and invasion of prostate cancer^46^. In addition, the CaMKKβ-AMPK pathway also plays a role in the signalling network that regulates adipocyte development^47^. However, the potential roles of LKB1^48^ and CaMKKβ^20^, two well-known activators of AMPK, as the upstream activators of AMPK in macrophages has not been established^49^. These studies and our results suggest multiple functions of the CaMKKβ-AMPK pathway in physiological and pathophysiological regulation.

Our study also demonstrated that NaHS protects mitochondrial function. This was shown by the increased expression of mitochondrial complex proteins and improved production of ATP in IR cells. Mitochondrial complexes are crucial for normal mitochondrial respiration and ATP production. It was reported that chicken mitochondria^50^ and mitochondria extracted from mammalian cells and tissues^51,52^ utilise H_2_S to maintain mitochondrial electron transport and to generate ATP. A study in streptozotocin-induced diabetic rats also showed an improved ATP production with H_2_S treatment^7^. In addition, our data demonstrated that the beneficial effects of H_2_S on mitochondria was diminished by the inhibitors of CamKKβ, AMPK and PI3K, which may suggest the important roles of the Ca^2+^-CaMKKβ-AMPK pathway and the PI3K/AKT pathway in maintaining mitochondrial function mediated by NaHS. The cAMP system in mitochondria functions tightly with the mitochondrial electron transport chain proteins^53^. In our study, it was demonstrated that cAMP was downregulated in the IR model and that NaHS upregulated cAMP levels. In parallel with the change in the expression of the mitochondrial complexes and mitochondrial biogenesis, the increase in cAMP was inhibited by the inhibitors of CamKKβ, AMPK and PI3K. Previous evidence showed that the mitochondrial cAMP system is tightly related to the phosphorylation of mitochondrial electron transport chain proteins and consequently related to the activity of proteins such as subunits of complexes I, II, III and cytochrome c oxidase^54^. Our data combined with that of others suggest that the Ca2+-CaMKKβ-AMPK and PI3K/AKT pathways are involved in the NaHS mediated protection of mitochondrial function and ATP production.

In conclusion, our study demonstrates that decreased endogenous H_2_S may be involved in the onset of insulin resistance induced by PA, However, exogenous NaHS attenuates PA-induced insulin resistance in C2C12 cells through the phosphorylation and activation of the insulin signalling pathway as well as the protection of mitochondrial function, which was at least partially mediated by the Ca^2+^-CaMKKβ-AMPK pathway and the PI3K/AKT pathway. This study may shed light on the pathogenesis of IR and provide a theoretical basis for treating diabetes in the clinic. This study was performed in an *in vitro* environment but not in an animal model. Thus, an *in vivo* work in the future to explore the role of NaHS on the insulin sensitivity and metabolism remains valuable.
